# The Effect of Treatment Strategy of Chronic Ischemic Mitral Regurgitation on Long-Term Outcomes in Coronary Artery Bypass Grafting

**DOI:** 10.21470/1678-9741-2017-0001

**Published:** 2017

**Authors:** Hüseyin Şaşkin, Kazım Serhan Ozcan, Mustafa İdiz

**Affiliations:** 1 Department of Cardiovascular Surgery of Derince Training and Research Hospital, Kocaeli, Turkey.; 2 Department of Cardiology of Derince Training and Research Hospital, Kocaeli, Turkey.; 3 Department of Cardiovascular Surgery of Acibadem İzmit Hospital, Kocaeli, Turkey.

**Keywords:** Coronary Artery Bypass, Mitral Valve Annuloplasty, Survival

## Abstract

**Objective:**

To investigate the mid- and long-term outcomes of case-based selective strategy of mitral ring annuloplasty during coronary artery bypass grafting in patients with coronary artery disease accompanied by chronic ischemic mitral regurgitation.

**Methods:**

132 patients who were diagnosed ischemic moderate to severe mitral regurgitation undergoing coronary artery bypass grafting in the same center with the same surgical team were divided into 2 groups and investigated retrospectively. Patients undergoing simultaneous mitral ring annuloplasty and coronary artery bypass grafting were enrolled to group 1 (n=58), patients undergoing isolated coronary artery bypass grafting were enrolled in group 2 (n=74).

**Results:**

The mean age of the patients were 65.0 ± 9.4 years and 39 (29.5%) of them were female. Preoperative New York Heart Association (NHYA) class (*P*=0.0001), atrial fibrillation (*P*=0.006) and the grade of mitral regurgitation (*P*=0.0001) were significantly different between the groups. Hospitalization for heart failure was required in 6 (10.6%) patients in group 1 and 19 (27.1%) patients in Group 2 (*P*=0.02). Hospital mortality and one-month postoperative mortality occurred in 2 (3.4%) patients in Group 1 and in 4 (5.4%) patients in Group 2 (*P*=0.69). Clinical follow-up was completed with 117 (88.6%) patients.

**Conclusion:**

Mitral ring annuloplasty in addition to the coronary artery bypass grafting is associated with improved NYHA functional class, increased ejection fraction, decreased residual mitral regurgitation. Further studies are needed to clarify the role of combined surgery on long-term outcomes. With proper tools and according to the decisions made by heart teams, both management strategies can be safely performed.

**Table:** 

Abbreviations, acronyms & symbols				
ACE	= Angiotensin-converting-enzyme		LVEDD	= Left ventricle end-diastolic diameter
ACT	= Activated clotting time		LVEDV	= Left ventricle end-diastolic volume
AF	= Atrial fibrillation		LVEF	= Left ventricular ejection fraction
CABG	= Coronary artery bypass grafting		LVESD	= Left ventricle end-systolic diameter
CPB	= Cardiopulmonary bypass		LVESV	= Left ventricle end-systolic volume
ECG	= Electrocardiography		MRA	= Mitral ring annuloplasty
EF	= Ejection fraction		NYHA	= New York Heart Association
EROA	= Effective regurgitant orifice area		PAP	= Pulmonary arterial pressure
IMI	= Ischemic mitral valve insufficiency		PISA	= Proximal isovelocity surface area
IMR	= Ischemic mitral regurgitation		RV	= Regurgitant volume
LIMA	= Left internal mammary artery		TTE	= Transthoracic echocardiography
LV	= Left ventricle			

## INTRODUCTION

Chronic ischemic mitral regurgitation (IMR) is an important complication of coronary artery disease and commonly accompanied by partial or total occlusion of one or two coronary arteries^[1]^. It is commonly associated with functional-valve incompetence due to myocardial injury and adverse left ventricular remodeling, which develops in approximately 50% of patients after an myocardial infarction, and moderate regurgitation occurs in more than 10% of patients^[2]^. IMR is associated with high morbidity and mortality rates, independent of treatment strategy^[3]^.

IMR is commonly accompanied with multivessel coronary artery disease needing surgical treatment. During coronary artery bypass grafting (CABG), mitral valve intervention is debated between cardiologists and cardiovascular surgeons and the treatment strategy were not clearly elucidated in the literature^[2]^. IMR is associated with poor outcomes in patients undergoing CABG but the outcome of mitral valve intervention during CABG is controversial^[4]^.

Deja et al.^[5]^ has observed that, in patients with moderate to severe mitral regurgitation in addition to left ventricle (LV) dysfunction, mitral repair in addition to CABG is associated with better survival compared to isolated CABG. In another study, it is observed that isolated CABG is associated with lower mortality in patients with ischemic mitral regurgitation^[3]^. In a study conducted in 355 patients with ischemic mitral regurgitation, Kim et al.^[6]^ observed that survival at 5 years was not different between isolated CABG and CABG in addition to mitral repair.

In this study we aimed to investigate the outcome of mitral repair in addition to CABG on mid- and long-term survival.

## METHODS

In this study, the data of a total of 1640 patients underwent to open cardiac surgery in a single center by the same surgical team between 2007 and 2014 were investigated retrospectively. Among these patients, 132 who had coronary artery disease diagnosed with cardiac catheterization (≥ 75% stenosis in at least one coronary artery) and moderate to severe (≥ 2 +) ischemic mitral valve insufficiency (IMI) diagnosed with echocardiography and left ventriculography, and undergoing CABG and/or mitral ring annuloplasty (MRA) were included in the study. Two groups were created: Group 1, which included patients who were operated for MRA together with CABG under cardiopulmonary bypass (CPB) (n=58); and Group 2, which included patients who were operated for isolated CABG under CPB (n=74).

Exclusion criteria included any echocardiographic evidence of structural (chordal or leaflet) mitral valve disease or ruptured papillary muscle. Likewise, patients who had acute IMI, reoperations for CABG, additional operations for diseases such as valvular, carotid and peripheral arterial diseases were excluded.

The controls of the patients included in the study were performed on the 6^th^ and 12^th^ postoperative months by the same clinician using transthoracic echocardiography (TTE). All of the patients' follow-up included measurements of the left ventricular ejection fraction (LVEF), left ventricle end-diastolic diameter (LVEDD), left ventricle end-systolic diameter (LVESD), left ventricle end-diastolic volume (LVEDV) and left ventricle end-systolic volume (LVESV) by Simpson method with TTE. The severity of IMI and evaluation of the mitral valve functions were done by visual method in all patients. Evaluation of the degree of IMI was performed by quantitative Doppler echocardiography as it is done the same as recently^[7]^. By quantitative Doppler echocardiography, measurements of stroke volume, regurgitant volume (RV) and effective regurgitant orifice area (EROA) using proximal isovelocity surface area (PISA) method were done. Grading the severity of IMI was classified on Table 1 in parallel with the literature. Intraoperative transesophageal echocardiography was performed during surgery in all patients and residual mitral regurgitation was evaluated after repair^[1]^. Patients who had moderate (Grade II), moderate to severe (Grade III) and severe IMI by echocardiography and clinical evaluation, but who had a short lifetime expectancy, high operative risk and severely low LVEF, were operated for isolated CABG operation with the decision of cardiology and cardiovascular surgery council. Patients whose mitral valves were not operated were added angiotensin-converting-enzyme (ACE) inhibitors and diuretics for medical treatment in the postoperative period.

**Table 1:** Grading of ischemic mitral regurgitation.

IMR degree	Regurgitant volume (mL)	Effective regurgitant orifice area (mm^2^)
I	< 30	< 20
II	30-44	20-29
III	45-59	30-39
IV	> 60	> 40

IMR=ischemic mitral regurgitation

The demographic and clinical data of the patients were obtained by using the software system of the hospital for records and archives to investigate the patient files, epicrisis, operation notes and laboratory results. Age, gender, smoking history, diabetes, hypertension, hyperlipidemia, LVEF, LVEDD, LVESD, LVESV, LVEDV, preoperative and postoperative laboratory parameters (hemoglobin, leukocyte count, thrombocyte count, fasting blood glucose, creatinine) operation information, the number of grafts used, duration of CPB and aortic cross-clamp, amount of blood products used and length of stay in the intensive care unit and hospital were recorded. In addition, New York Heart Association (NYHA) functional class was analyzed.

Hypertension was accepted as a blood pressure ≥ 140/90 mmHg or usage of antihypertensive drugs; smoking was accepted positive if the patient had not quitted smoking for the last one year. Diabetes was accepted as fasting blood glucose ≥ 126 mg/dL or use of antidiabetic drugs, hyperlipidemia was accepted as total cholesterol > 220 mg/dL and LDL-cholesterol > 130 mg/dL or use of antihyperlipidemic drugs.

All of the patients were transferred to intensive care unit intubated. They were extubated following onset of spontaneous breathing and normalization of orientation and cooperation if the haemodynamic and respiratory functions were appropriate. If there was no contraindication, 50 mg/day of metoprolol was started orally to all patients following the 1^st^ postoperative day. The diagnosis of postoperative atrial fibrillation (AF) was made by standard 12 derivation electrocardiography (ECG). Mortality during the stay in the hospital following operation or in the first 30 postoperative days was accepted as postoperative early-term mortality.

Functional status was assessed according to NYHA criteria during follow-up. Clinical follow-up data were collected during patient visits to the department or by telephone interviews. Operative mortality was defined as death within 30 days of the index procedure or before discharge. The authors had full access to the data and take full responsibility for its integrity. All authors have read and agree to the manuscript as written.

Written informed consent form was obtained from all the patients included in the study. This study complied with the Declaration of Helsinki and was carried out following approval of Ethics Committee for Clinical Trials of Medical Faculty of Kocaeli University.

### Operative Technique

Median sternotomy was applied following the routine anesthesia application in the surgery. Bypass grafts (saphenous vein and left internal mammary artery) were prepared. Systemic heparinization was ensured by administration of 300 IU/kg heparin in a fashion that activated clotting time (ACT) was greater than 450 seconds. CPB was performed by clinic aortic arterial-bicaval venous cannulation. Two-stage cannula was used for venous cannulation for patients who were not applied mitral ring annuloplasty. In all patients, non-pulsatile roller pump and membrane oxygenator were used for CPB. Surgical procedures were established in moderate systemic hypothermia (28-30ºC). CPB was applied in a fashion that flow rate would be 2.2 to 2.5 L/min/m^2^; the mean perfusion pressure would be between 50 and 80 mmHg, hematocrit values would be between 20% and 25%. Myocardial protection was achieved via antegrade hypothermic and hyperpotasemic blood cardioplegic arrest, followed by continuous administration of retrograde blood cardioplegic solution for the duration of cross-clamping. After cross-clamp application, the initial distal saphenous vein anastomoses were performed. This step was followed consecutively by ring implantation, distal anastomosis of the left internal mammary artery (LIMA). Cross-clamps were removed after de-airing of the heart. All proximal anastomoses were done in the heart working under partial clamp in all patients operated on.

Mitral ring annuloplasty was performed in patients in whom the mitral leaflets could be coapted. A circular SJM Tailor^TM^ Flexible Ring (St. Jude Medical, Inc. St. Paul, USA) was used in all instances. The appropriate ring size was determined from measurement of the anterior leaflet. Ring sizes 28 to 32 were used. In each patient, 12 TiCron^TM^ 2-0 sutures (Covidien Syneture; Mansfield, MA, USA) were placed. After CPB was stopped, intraoperative transesophageal echocardiography was performed in order to rule out substantial valvular insufficiency. In our study, no patient required mitral reintervention.

### Statistical Analysis

Statistical analysis was performed using the SPSS software version 12.0 (SPSS Inc, Chicago, IL, USA). Among the data measured, those showing normal distribution were expressed as mean ± standard deviation; those that do not show normal distribution were expressed as median (minimum-maximum). The data obtained by counting were given as percentages (%). Among the data measured, the normality of distribution was evaluated by histogram or Kolmogorov-Smirnov test, the homogeneity of distribution was evaluated by the Levene's test for equality of variance. Among the data measured, the difference between the groups was evaluated by Student's t-test in normal and homogenous distribution and by Mann-Whitney U test in a distribution that is not normal and homogenous. Among the data obtained by counting, the differences between the groups were evaluated by parametric or non-parametric Pearson's chi-square test or Fisher's exact test according to the distribution being parametric or not. Survival curves were constructed for each group using the Kaplan-Meier method, and comparisons were made using the log-rank test. A *P* value < 0.05 was considered statistically significant.

## RESULTS

The demographic characteristics and clinical data of the patients were summarized in Table 2. Preoperative NHYA class (*P*=0.0001), AF (*P*=0.006) and the grade of mitral regurgitation (*P*=0.0001) were significantly different between groups. The preoperative blood analysis and hematological parameters of the patients summarized in Table 3. No significant differences in preoperative blood analysis and hematological parameters were found between the groups.

**Table 2:** Evaluation of groups for preoperative characteristics.

Variables	CABG + mitral annuloplasty Group 1 (n=58)	Isolated CABG Group 2 (n=74)	*P*
Age, mean±SD, year	64.10±8.74	65.66±9.95	0.22***
Gender, n (%)			
Male	40 (69.0%)	53 (71.6%)	0.74*
Female	18 (31.0%)	21 (28.4%)	
EuroSCORE (st), mean±SD	7.21±1.77	6.93±1.14	0.73***
PAP (mmHg), mean±SD	42.59±5.15	41.01±4.96	0.11***
NYHA class, mean±SD	3.55±0.50	2.46±0.62	0.0001***
COPD, n (%)	10 (17.2%)	9 (9.5%)	0.19*
Left main lesion > 50%, n (%)	8 (13.8%)	13 (17.6)	0.56*
Rhythm, n (%)			
Sinus rhythm	47 (81.0%)	71 (95.9%)	0.006*
Atrial fibrillation rhythm	11 (19.0%)	3 (4.1%)	
Diabetes mellitus, n (%)	27 (46.6%)	31 (41.9%)	0.59*
Hyperlipidemia, n (%)	23 (39.7%)	31 (41.9%)	0.80*
Hypertension, n (%)	27 (46.6%)	40 (54.1%)	0.39*
Smoking, n (%)	20 (34.5%)	27 (36.5%)	0.81*
Previous neurological event, n (%)	3 (5.2%)	5 (6.8%)	0.98*
Unstable angina, n (%)	21 (36.2%)	31 (41.9%)	0.51*
Severity of mitral regurgitation, n (%)			
Moderate	16 (27.6%)	47 (63.5%)	0.0001*
Severe	42 (72.4%)	27 (36.5%)	

COPD=chronic obstructive pulmonary disease; NYHA=New York Heart Association; PAP=pulmonary artery pressure

* Pearson's chi-square test or Fisher's exact test.

** Student's-t test.

*** Mann-Whitney U test.

**Table 3:** Preoperative blood results and haematological parameters of patients.

Preoperative blood results and haematological parameters	CABG + mitral annuloplasty Group 1 (n=58) Median (min-max)	Isolated CABG Group 2 (n=74) Median (min-max)	*P* value
Haemoglobin (mg/dL)	13.6 (10.4-15.5)	14.0 (10.4-16.5)	0.36**
Haematocrit (%)	40.7 (30.6-48.1)	42.6 (30.5-48.9)	0.31**
Creatinine (mg/dL)	0.90 (0.56-1.83)	0.90 (0.50-1.89)	0.64**
Urea (mg/dL)	41 (31-68)	43 (31-49)	0.82**
Leukocyte count (x103/µL)	7.75 (5.90-9.70)	7.60 (5.10-10.20)	0.92**
Thrombocyte count (x103/µL)	261 (180-401)	255 (147-422)	0.41**
C-reactive protein (mg/L)	0.55 (0.17-1.76)	0.54 (0.16-1.87)	0.97**

** Mann-Whitney U test. CABG=coronary artery bypass grafting

The intraoperative and postoperative data of the patients were shown in Table 4. Aortic cross-clamp time (*P*=0.0001), CBP time (*P*=0.0001), intubation time (*P*=0.0001), use of inotropic support (*P*=0.004) and length of hospital stay (*P*=0.01) presence were significantly different between the groups. The average number of distal anastomoses was 3.53±0.60 in Group 1, and 3.54±0.86 in Group 2, which was not statistically different between the groups (*P*=0.92). LIMA was used in 55 (94.8%) patients in Group 1 and 68 (91.9%) patients in Group 2.

**Table 4:** Preoperative blood results and haematological parameters of patients.

Variables	CABG + mitral annuloplasty Group 1 (n=58) Mean±SD	Isolated CABG Group 2 (n=74) Mean±SD	*P*
CCT, minute	90.29±6.99	59.00±10.62	0.0001**
CPB time, minute	132.09±6.64	91.19±11.69	0.0001**
Intubation time, hours	8.00±5.06	6.71±6.12	0.0001**
Amount of drainage, mL	382.7±211.6	367.6±192.2	0.51**
Number of distal anastomoses	3.53±0.60	3.54±0.86	0.92**
Length of intensive care unit stay, hours	37.74±31.33	30.01±28.09	0.15**
Length of hospital stay, days	7.17±3.16	5.85±1.37	0.01**
Use of inotropic support, n (%)	18 (31.0%)	8 (10.8%)	0.004**
Use of blood products, n (%)	32 (55.2%)	30 (40.5%)	0.10*

CABG=coronary artery bypass grafting; CCT=cross-clamp time; CPB=cardiopulmonary bypass

* Pearson's chi-square test or Fisher's exact test.

** Mann-Whitney U test.

Evaluations of the early postoperative complications were summarized in Table 5. Postoperative AF was significantly different between groups (*P*=0.005) and other parameters were not significantly different between the groups. There were 6 hospitalizations for heart failure in Group 1 (10.6%) and 19 in Group 2 (27.1%) (*P*=0.02). Mortality in the hospital and in the 1^st^ postoperative month occurred in 2 (3.4%) patients in Group 1 and in 4 (5.4%) patients in Group 2, which was not statistically different between the groups (*P*=0.69). The causes of operative mortality were low cardiac output syndrome in 3 patients and multiple organ failure, mediastinitis and pneumonia in one patient each.

**Table 5:** Evaluation of the early postoperative complications.

Complications	CABG + mitral annuloplasty Group 1 (n=58)	Isolated CABG Group 2 (n=74)	*P*
Neurological events, n (%)	3 (5.2%)	5 (6.8%)	1.00*
Respiratory event, n (%)	10 (17.2%)	16 (21.6%)	0.53*
Renal disorder, n (%)	2 (3.4%)	4 (%.4%)	0.69*
IABP, n (%)	6 (10.3%)	5 (6.8%)	0.53*
Bleeding revision, n (%)	3 (5.2%)	2 (2.7%)	0.65*
Sternal infection, n (%)	5 (8.6%)	4 (5.4%)	0.51*
Sternal revision, n (%)	3 (5.2%)	2 (2.7%)	0.65*
Postoperative new-onset AF, n (%)	22 (37.9%)	12 (16.2%)	0.005*

AF=atrial fibrillation; CABG=coronary artery bypass grafting; IABP=intra-aortic balloon pump

* Pearson's chi-square test or Fisher's exact test.

Clinical follow-up was completed with 117 (88.6%) patients, with a mean follow-up period of 51.3 ± 26.8 months. There were 3 deaths in the CABG plus mitral ring annuloplasty group over a mean follow-up of 45.9 ± 26.0 months, yielding an estimated actuarial 8-year survival rate of 90.4% ± 4.1%. NYHA class in this group improved from 3.6 ± 0.5 to 1.3 ± 0.5 during follow-up. There were 6 deaths in the isolated CABG group over a mean follow-up of 55.6 ± 26.8 months, yielding an estimated actuarial 8-year survival rate of 84.1%±4.7%. NYHA class in this group improved from 2.5 ± 0.6 to 2.1 ± 0.7 during follow-up.

**Fig. 1:**
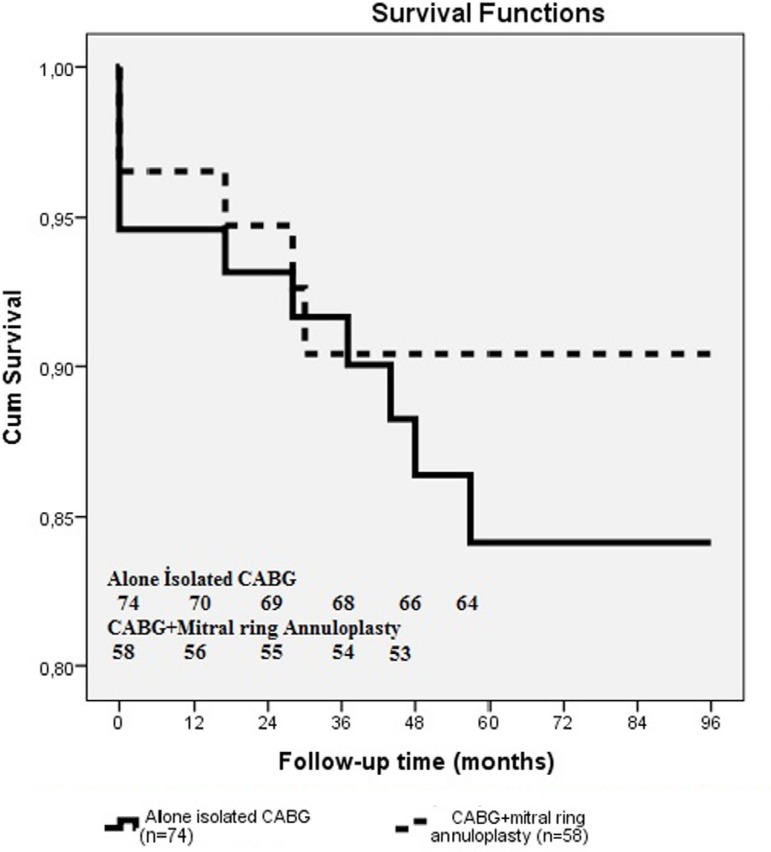
Kaplan-Meier survival curves of our patients undergone isolated CABG and CABG plus MRA.

Long-term survival was not different between groups (*P*=0.56, 95% CI: 91.64 (88.23-95.05) (Figure 1). Preoperative and postoperative NYHA class were significantly different between the groups (*P*=0.0001).

Follow-up TTE was performed on all surviving patients 6 months and 1 year after surgery. IMR was improved in 51 (92.7%) patients in the MRA plus CABG group, compared with 47 (67.1%) patients in the CABG alone group, which was statistically different between the groups (*P*=0.001). In the echocardiographic evaluation of survived patients with severe mitral regurgitation, 39 (95.1%) patients in Group 1 and 13 (56.5%) patients in Group 2 had reduction in the degree of mitral regurgitation (*P*=0.0001) in the 1-year follow-up.

In the echocardiographic evaluation of survived patients with severe moderate regurgitation, 11 (84.6%) patients in Group 1 and 24 (51.1%) patients in Group 2 had reduction in the degree of mitral regurgitation (*P*=0.003) in the 1-year follow-up.

The echocardiographic data of the survived patients (preoperative, 6^th^ and 12^th^ postoperative months) and comparison of the echocardiographic data in groups and between the groups were depicted in Table 6. Postoperative ejection fraction (EF) was significantly improved in Group 1 and was not significant in Group 2 (*P*=0.0001 for Group 1, *P*=0.22 for Group 2).

**Table 6:** Comparison of intragroup and intergroup for preoperative and 6th and 12th postoperative months echocardiographic data.

Variables	CABG + mitral annuloplasty Group 1 (n=58) Median (min-max)	Isolated CABG Group 2 (n=74) Median (min-max)	*P*
Preoperative EF (%)	40 (25-50)	40 (20-55)	0.19**
Postoperative 6 months EF (%)	45 (30-55)	40 (20-55)	0.001**
Postoperative 12 months EF (%)	45 (35-60)	40 (20-55)	0.0001**
*P* value	0.0001*	0.22*	
Preoperative LVESD (mm)	49 (45-54)	46 (42-51)	0.0001**
Postoperative 6 months LVESD (mm)	46 (43-51)	45 (42-51)	0.001**
Postoperative 12 months LVESD (mm)	43 (40-48)	45 (42-50)	0.0001**
*P* value	0.0001*	0.001*	
Preoperative LVEDD (mm)	60 (56-65)	57 (53-62)	0.0001**
Postoperative 6 months LVEDD (mm)	57 (54-62)	57 (54-61)	0.18**
Postoperative 12 months LVEDD (mm)	54 (50-58)	56 (53-61)	0.0001**
*P* value	0.0001*	0.11*	
Preoperative LVESV (mm)	91 (83-97)	84 (76-92)	0.0001**
Postoperative 6 months LVESV (mm)	73 (67-84)	84.5 (75-91)	0.0001**
Postoperative 12 months LVESV (mm)	61 (54-75)	85 (75-94)	0.0001**
*P* value	0.0001*	0.43*	
Preoperative LVEDV (mL)	137 (125-145)	128 (117-137)	0.0001**
Postoperative 6 months LVEDV (mL)	121 (112-125)	127 (120-138)	0.0001**
Postoperative 12 months LVEDV (mL)	109 (102-116)	127 (120-140)	0.0001**
*P* value	0.0001*	0.16*	
Preoperative EROA (mm^2^)	42 (32-48)	28 (24-42)	0.0001**
Postoperative 6 months EROA (mm^2^)	21 (18-23)	29 (25-43)	0.0001**
Postoperative 12 months EROA (mm^2^)	18 (16-20)	30 (25-46)	0.0001**
*P* value	0.0001*	0.0001*	
Preoperative RV (mL)	63 (45-73)	40 (32-62)	0.0001**
Postoperative 6 months RV (mL)	33 (23-42)	40 (28-58)	0.0001**
Postoperative 12 months RV (mL)	20 (17-29)	42 ( 25-63)	0.0001**
*P* value	0.0001*	0.005*	

CABG=coronary artery bypass grafting; EF=ejection fraction; LVED=left ventricle end-diastolic diameter; LVEF=left ventricular ejection fraction; LVESD=left ventricle end-systolic diameter; LVEDV=left ventricle end-diastolic volume; LVESV=left ventricle endsystolic volume; RV=regurgitant volume; EROA=effective regurgitant orifice area

* Friedman test.

** Mann-Whitney U test.

Postoperative LVESD, LVEDD, LVESV, LVEDV, RV and EROA were significantly decreased in Group 1. LVESD was significantly decreased in Group 1 (*P*=0.001), EROA (*P*=0.0001) and RV dimensions (*P*=0.005) was significantly decreased in Group 2.

## DISCUSSION

We evaluated the efficacy of concomitant CABG plus mitral ring annuloplasty (MRA) compared with CABG alone in patients with moderate and severe IMR. In the present study, we found that combined CABG and MRA in patients with moderate and severe IMR resulted in a greater decrease of early postoperative mitral regurgitation than CABG alone. We have observed that, in addition to the CABG, MRA is associated with improved functional capacity. Patients in both groups had low early and late mortality rates, despite the presence of impaired LVEF and moderate and severe MR. We have observed that early-, mid- and long-term mortality was not significantly different between the groups.

Mitral repair in addition to CABG was associated with better NHYA functional class and improvement in EF and decrease in LVESD, LVEDD, LVESV, LVEDV, EROA and regurgitation volume.

Approximately 20% of MR is ischemic and associated with myocardial infarction^[8]^. IMR is an important complication of myocardial infarction and observed in 40% of patients with this condition. Regurgitation is caused by annular dilation and papillary muscle displacement in an anatomically normal valve^[9]^.

IMR associated with coronary artery disease can occur in an acute or chronic fashion^[10]^. Acute ischemic mitral regurgitation was an exclusion criteria in our study.

Chronic IMR is still a significant clinical problem. It is present in 10-20% of patients with coronary artery disease and is associated with a worse prognosis after myocardial infarction and subsequent revascularization. Currently, CABG combined with restrictive annuloplasty is the most commonly performed surgical procedure^[11]^.

Chronic mitral regurgitation is commonly accompanied with left ventricular segmentary wall motion abnormality in one or more LV wall with occlusion or stenosis of the culprit vessel which occurs 16 days after acute myocardial infarction^[12]^.

Mitral regurgitation is graded mild, moderate or severe based on echocardiographic and ventriculography criteria^[13]^. EROA criteria for severe mitral regurgitation was 0.4 cm^2^ and RV criteria was 50 mL in patients without ischemia, EROA criteria for severe mitral regurgitation was 0.2 cm^2^ and RV criteria was 30 mL in patients with ischemia^[14]^. We have performed MRA in patients with EROA 0.2 cm^2^ and RV 30 mL in our study.

The presence of IMR in addition to coronary artery disease requiring surgical revascularization is an important topic for both cardiologists and cardiovascular surgeons. The surgical options for moderate to severe IMR are mitral repair or replacement in addition to CABG or isolated CABG^[15]^.

The patient's symptoms, the severity of mitral regurgitation, repairability of the mitral valve, ischemic burden and surgical risk are considered for surgical intervention^[16]^.

The surgical options for IMR and the results of surgery are controversial in the literature^[9]^.

Wong et al.^[17]^ has investigated the role of MRA in addition to CABG in patients with moderate to severe mitral regurgitation. In the long-term follow-up the mortality rate for MRA was not different between the groups, but the degree of mitral regurgitation was significantly decreased in MRA group.

Three hundred ninety patients with moderate to severe IMR were involved in a study and the groups were compared for MRA in addition to CABG. MRA group was associated with lesser degree of mitral regurgitation and less symptomatic in short-term follow-up, but in the long-term functional class and survival were not different between groups^[18]^. In our study we have observed better NYHA functional status and lesser degree of mitral regurgitation in the MRA group, but in the long-term follow-up survival was not different between the groups.

Surgical timing is another important issue, because early intervention can prevent the irreversible myopathic changes consequent to remodeling^[19]^. Fattouch et al.^[20]^ randomized 102 patients with moderate mitral regurgitation to CABG or CABG plus mitral repair. CABG plus mitral repair was associated with decreased LVESD, LVEDD, pulmonary arterial pressure (PAP) and left atrial size. Similar to their findings we have observed decreased LVESD, LVEDD, LVESV, LVEDV, PAP, EROA, regurgitation volume and MR degree in patients with CABG plus MRA.

The presence of MR has been associated with adverse cardiac events and mortality^[8]^. Isolated CABG is usually performed in moderate and moderate-to-severe mitral regurgitation in high-risk patients with poor general performance. Percutaneous mitral repair may be an option for these patients. On the other hand, in patients with an acceptable risk profile, mitral repair is performed in IMR in the majority of patients^[21,22]^. We have performed isolated CABG in patients with high surgical risk and poor perioperative state.

Mallidi et al.^[23]^ observed higher rate of heart failure and shorter cardiac event free survival in CABG-only patients who had mild-to-moderate mitral regurgitation, in comparison with patients who had no regurgitation.

In contrast to this finding Silberman et al.^[24]^ has observed higher rate of heart failure in patients with CABG plus mitral repair in 231 patients. Smith et al.^[2]^ has involved 301 patients with IMR to their study and observed that hospitalization for heart failure was similar between isolated CABG and CABG plus mitral repair groups (13.2% and 14.7%, respectively). In our study we have observed that hospitalization for heart failure was significantly higher in patients with isolated CABG group.

The hospital mortality rate of CABG-only patients who have no mitral regurgitation ranges from 0 to 6.9% and the rate for CABG-only patients with moderate mitral regurgitation ranges from 1.8% to 12%^[25]^. Harris et al.^[26]^ has investigated the role of mitral intervention in 176 patients with moderate IMR and the mortality rate was 9% in CABG group and 21% in mitral intervention group (*P*=0.047). Silberman et al.^[24]^ has investigated the role of MRA in addition to CABG in patients with moderate IMR and have observed similar mortality rates (7%) between the groups. In our study, we have observed similar mortality rates in patients with CABG alone and CABG plus MRA (3.4% and 5.4%, respectively).

### Limitations

A few limitations of our study deserve mention. This is a single centre retrospective study. Another limitation is the echocardiographic evaluation of MR grade and the lack of complete follow-up. Small sample size, especially in the propensity analysis, is another limitation of this study. The issue of myocardial viability is also important in surgical management of IMR. Improvement in the grade of mitral regurgitation with CABG is associated with functional improvement of dysfunctional but viable myocardium. Viability studies may have a role for prediction of improvement in mitral regurgitation. We did not routinely perform viability testing. In addition, we did not examine the relation between viability test results and improvements in IMR.

## CONCLUSION

Patients who have undergone either CABG alone or CABG plus MRA surgery have experienced very low early and late mortality rates, despite the presence of multiple comorbidities, impaired LVEF, and moderate and severe MR. Mitral ring annuloplasty can be performed safely, concomitantly with CABG, in patients who have moderate and severe IMR. In such patients, the combined procedure resulted in a greater decrease in early postoperative MR, LVESD, LVEDD, LVESV, LVEDV and EROA than CABG alone.

MRA in addition to CABG is associated with improvement in NHYA functional class. Prospective studies in a randomized fashion are needed to better define the role and outcome of MRA in this population.

**Table:** 

Authors' roles & responsibilities	
HS	Substantial contributions to the conception or design of the work; or the acquisition, analysis, or interpretation of data for the work; final approval of the version to be published
KSO	Agreement to be accountable for all aspects of the work in ensuring that questions related to the accuracy or integrity of any part of the work are appropriately investigated and resolved; final approval of the version to be published
MI	Substantial contributions to the conception or design of the work; or the acquisition, analysis, or interpretation of data for the work; final approval of the version to be published
